# Radiation therapy for epithelial ovarian cancer brain metastases: clinical outcomes and predictors of survival

**DOI:** 10.1186/1748-717X-8-36

**Published:** 2013-02-15

**Authors:** Sewit Teckie, Vicky Makker, Viviane Tabar, Kaled Alektiar, Carol Aghajanian, Martee Hensley, Kathryn Beal

**Affiliations:** 1 Department of Radiation Oncology, Memorial Sloan-Kettering Cancer Center, 1275 York Avenue, New York, NY, USA; 2 Department of Medicine, Memorial Sloan-Kettering Cancer Center and Weill Cornell Medical College, 1275 York Avenue, New York, NY, USA; 3 Department of Neurosurgery, Memorial Sloan-Kettering Cancer Center, 1275 York Avenue, New York, NY, USA

**Keywords:** Ovarian cancer, Brain metastases, Leptomeningeal disease, Palliation

## Abstract

**Background:**

Brain metastases (BM) and leptomeningeal disease (LMD) are uncommon in epithelial ovarian cancer (EOC). We investigate the outcomes of modern radiation therapy (RT) as a primary treatment modality in patients with EOC BM and LMD.

**Methods:**

We evaluated 60 patients with EOC treated at our institution from 1996 to 2010 who developed BM. All information was obtained from chart review.

**Results:**

At EOC diagnosis, median age was 56.1 years and 88% of patients were stage III-IV. At time of BM diagnosis, 46.7% of patients had 1 BM, 16.7% had two to three, 26.7% had four or more, and 10% had LMD. Median follow-up after BM was 9.3 months (range, 0.3-82.3). All patients received RT, and 37% had surgical resection. LMD occurred in the primary or recurrent setting in 12 patients (20%), 9 of whom received RT. Median overall survival (OS) after BM was 9.7 months for all patients (95% CI 5.9–13.5), and 16.1 months (95% CI 3.8-28.3) in patients with one BM. On multivariate analysis, Karnofsky performance status less than 70 (hazard ratio [HR] 2.86, *p* = 0.018), four or more BM (HR 3.18, *p* = 0.05), LMD (HR 8.22, *p* = 0.013), and uncontrolled primary tumor (HR 2.84, *p* = 0.008) were significantly associated with inferior OS. Use of surgery was not significant (*p* = 0.31). Median central nervous system freedom from progression (CNS-FFP) in 47 patients with follow-up was 18.5 months (95% CI, 9.3–27.9). Only four or more BM (HR 2.56, *p* = 0.04) was significantly associated with poorer CNS-FFP.

**Conclusions:**

Based on our results, RT appears to be an effective treatment modality for brain metastases from EOC and should be routinely offered. Karnofsky performance status less than 70, four or more BM, LMD, and uncontrolled primary tumor predict for worse survival after RT for EOC BM. Whether RT is superior to surgery or chemotherapy for EOC BM remains to be seen in a larger cohort.

## Background

Epithelial ovarian cancer (EOC) accounts for 3% of cancers among women, but is the fifth leading cause of cancer death in women and the leading cause of gynecologic cancer death [1]. The predominant form of relapse after primary surgery and chemotherapy for EOC is in the abdomen and pelvis [2]. Central nervous system (CNS) and brain metastases (BM) in these patients are a rare occurrence, with reported incidence of 0.29-11.6% [3-9], but may be increasing in incidence as extracranial disease is better controlled with improved surgical and chemotherapeutic options [9-11].

The therapeutic approach to patients with BM from EOC is challenging due to the small numbers of cases and short follow-up periods available in other series [3-10,12]. No studies investigate the impact of modern radiation therapy (RT) as a primary treatment modality in these patients. Treatments vary widely, including best supportive care, chemotherapy, steroids, whole brain radiation therapy (WBRT), surgical resection, and stereotactic radiosurgery (SRS). Median survival in existing studies of EOC with BM has generally been poor, on the order of several months, but some studies report survival as high as 18–33 months in selected patients treated with multimodality therapy that combines surgery, radiation therapy, and systemic chemotherapy [3,5,13,14]. Prior studies of BM in advanced malignancies and in small series of EOC have found performance status, age, primary tumor control, extracranial metastases, and treatment modality for BM to be predictors of survival after BM [4,11,12,15,16]. Leptomeningeal disease (LMD) is regarded as a factor for poor prognosis in other metastatic cancers [17-19] and has been observed with increasing incidence in advanced malignancies, especially in the era of magnetic resonance imaging (MRI) [18]. However, LMD is still rarely reported in EOC.

We reviewed our institution’s experience using modern RT, with or without craniotomy, to treat patients with BM and LMD from EOC. We also identify predictors of survival after RT in this patient population.

## Methods

This study was approved by the Institutional Review Board, who also approved a waiver of informed consent. Our institution’s gynecology database was searched for patients with EOC who developed BM and received RT. We identified 60 patients who were diagnosed between October 1996 and April 2010. We performed a retrospective chart review to obtain demographic data, details of initial EOC diagnosis and treatment, Karnofsky Performance Status (KPS), stage, grade, date of BM diagnosis, interval to BM, site and number of BM, treatment type for BM, systemic disease at BM diagnosis, follow-up and response to treatment, date of CNS relapse or recurrence, time interval to relapse or recurrence after initial BM, and date of death or last follow-up. For two patients, the date of death could not be determined. These patients were censored at date of last follow-up. All BM, including LMD, were diagnosed by imaging, most commonly MRI.

Overall survival (OS) was calculated as the time from initial EOC or BM diagnosis to date of death or last follow-up. CNS freedom from progression (CNS-FFP) after BM was calculated from date of BM diagnosis to date of CNS recurrence or last follow-up imaging. Patients without follow-up imaging after BM treatment were excluded from the CNS-FFP analysis. Survival rates were determined using the Kaplan-Meier method, and survival curves were compared using the log-rank test. Univariate (UVA) and multivariate (MVA) survival analyses were performed using a Cox proportional hazards model on OS and CNS-FFP. Variables with *p* value ≤0.05 by UVA were considered for the MVA, and forward procedure was used to build the final model. A *p* value ≤0.05 was considered significant for all analyses. All statistical analysis was accomplished with the SPSS software package, version 19 (IBM, Armonk, NY). 

## Results

### Clinical characteristics

Patient characteristics at the time of initial BM diagnosis are listed in Table 1. Median age at diagnosis of EOC was 56.1 years (range, 31.2-79.0). Stage distribution [20] at original diagnosis of EOC was 3 patients with stage I (5%), 4 with stage II (6.7%), 40 with stage III (66.7%), and 13 with stage IV (21.7%). Histologic grade at diagnosis was 2 patients with grade 1 (3%), 7 with grade 2 (12%), 49 with grade 3 (82%), and 2 unknown (3%). Tumor histology was distributed as follows: 42 (70%) papillary serous, 8 (13%) endometrioid, 3 (5%) adenocarcinoma not otherwise specified, 2 (3%) mixed carcinoma, and 1 each of mixed adenocarcinoma, clear cell carcinoma, mucinous adenocarcinoma, small cell carcinoma, and cystic ovarian carcinoma.

**Table 1:** Clinical characteristics at time of initial brain metastases

**Characteristics**	**N**	**Range**
Median age at BM Dx	56.1 years	31.2-79.0
Median interval from EOC Dx to BM	3.4 years	0.08-26.8
Median interval from EOC Dx to BM in stage I-II EOC	5.9 years	3.7-10.8
Median interval from EOC Dx to BM in stage III-IV EOC	3.1 years	0.08-26.8
	**Number**	**Percent**
Age <65	52	87%
Age ≥65	8	13%
KPS <70	9	15%
KPS ≥70	42	70%
KPS unknown	9	15%
Number of BM at initial BM Dx:		
1	28	46.7%
2-3	10	16.7%
≥4	16	26.7%
LMD	6	10%
Location of initial BM:		
Cerebral hemispheres	29	48%
Cerebellum	10	17%
Both	21	35%
Extracranial metastases at time of BM Dx:		
None	10	17%
Limited and stable	11	18%
Limited and progressive	11	18%
Extensive and stable	11	18%
Extensive and progressive	16	27%
Unknown	1	2%
Primary tumor status (abdomen):		
Controlled	36	60%
Uncontrolled	23	38%
Unknown	1	2%
Chemotherapy in 3 months preceding BM Dx:		
Yes	37	62%
No	23	38%

BM = brain metastasis; Dx = diagnosis; EOC = epithelial ovarian cancer; KPS = Karnofsky Performance Score; LMD = leptomeningeal disease.

Median follow-up from BM diagnosis for all 60 patients was 9.3 months (range, 0.3-82.3). Median follow-up from BM diagnosis for the six patients alive at analysis was 27.1 months (range, 0.7-82.2).

### Initial treatment of brain metastases

**Figure 1:**
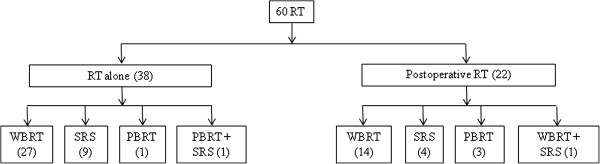
Treatments for initial brain metastases.

Treatments for initial BM are shown in Figure 1. RT was the sole treatment in 38 patients. Twenty-two patients were also treated with surgical resection for initial BM: 16 had a single BM, three had 2–3 BM, and three had four or more BM. In the six patients who underwent craniotomy and had multiple BM, four patients had only the one most symptomatic lesion removed, and two patients who presented with two lesions had both completely resected. Median WBRT dose was 3000 cGy (range, 600 cGy-4400 cGy). Median SRS dose was 2100 cGy (range, 1400–2200 cGy). Thirty-six patients (60%) had at least one cycle of systemic chemotherapy following RT ± surgery. After brain metastasis, ovarian cancers were treated with vastly heterogenous systemic regimens: we counted 18 unique regimens, and 19 patients received two or more lines of chemotherapy. The most common combination regimens were carboplatin and paclitaxel, followed by gemcitabine and carboplatin or cisplatin. The most common single-agent regimens were paclitaxel, gemcitabine, carboplatin, and liposomal doxorubicin. Patients with a KPS ≥70 were more likely than patients with KPS <70 to receive systemic therapy following local treatment of brain disease (Chi square, *p* = 0.04). The remaining 40% of patients either did not have information on chemotherapy available, or did not receive chemotherapy. This group had a median OS of 2.4 months after completing RT (range, 0.1-81.7).

### Recurrences and salvage RT

Six patients were alive at time of analysis at a median follow-up of 27.1 months after their initial BM (range, 0.7-82.2). Twenty-four patients developed recurrent or progressive CNS disease. Median time to recurrence was 7.3 months (range, 0.9-46.3). Thirteen patients received further RT for their recurrence, including three treated with conventional RT to the spine for leptomeningeal recurrence. Seven patients received further systemic chemotherapy following diagnosis of their recurrent CNS disease.

### Leptomeningeal disease

LMD was diagnosed in 12 (20%) patients, either at time of initial BM (n = 6) or as relapse of CNS disease (n = 6). Median interval from initial BM diagnosis to secondary LMD diagnosis was 7.2 months (range, 2.5-44.5). All but one patient with LMD had a preceding or synchronous BM. Treatments for LMD in the primary setting included five WBRT and one partial brain radiation therapy (PBRT). Treatments for relapsed LMD included WBRT (n = 2), spine RT (2), systemic chemotherapy (1), and best supportive care (1). All patients who received RT and had follow-up imaging had at least a partial response to therapy.

### Survival

**Figure 2:**
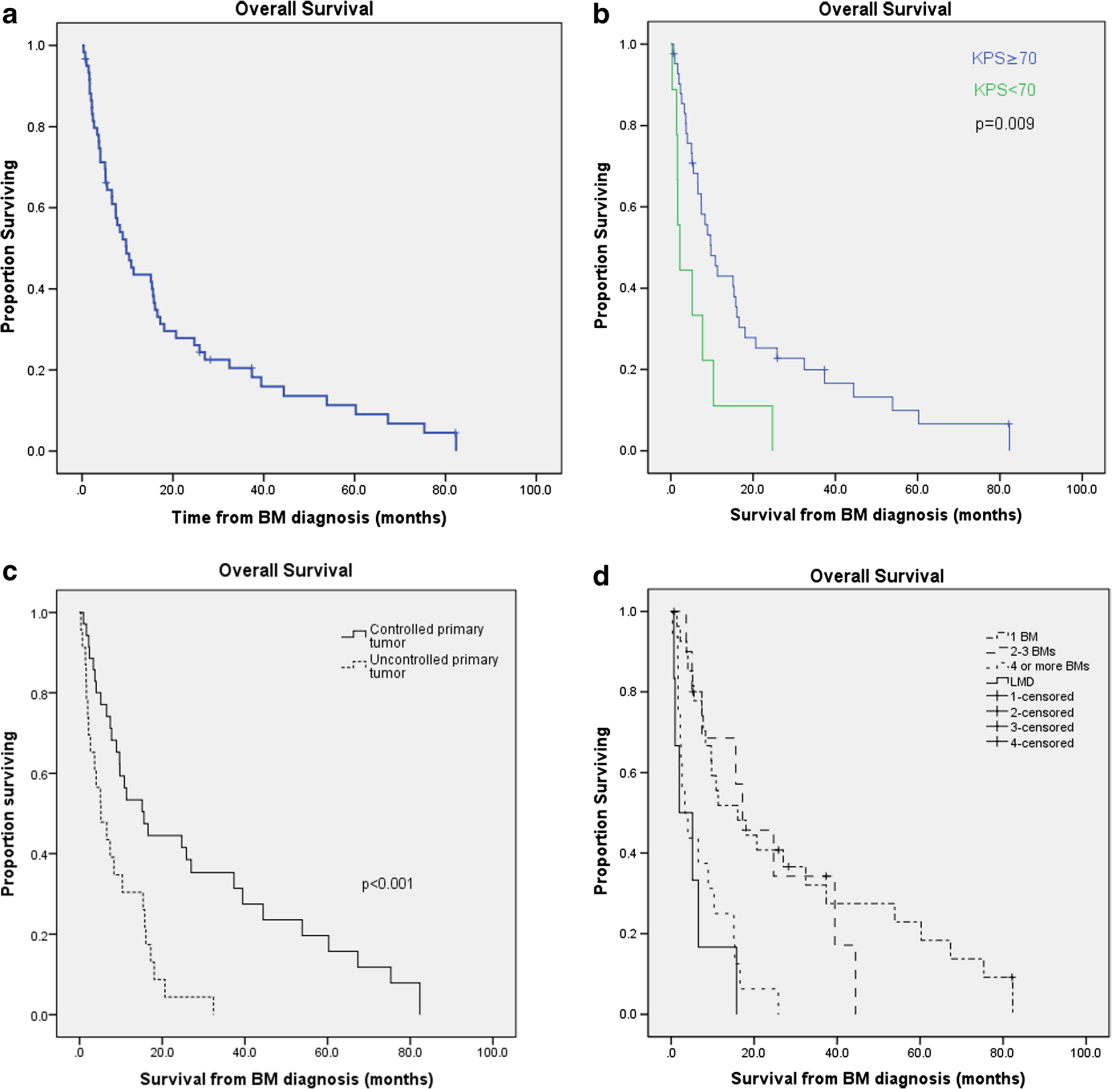
**a**. **Overall survival for all patients using Kaplan-Meier method. b**. Overall survival stratified by Karnofsky performance status. **c**. Overall survival stratified by primary tumor control status. **d**. Overall survival stratified by number of brain metastases and leptomeningeal disease.

Median OS from EOC diagnosis was 67.1 months (95% CI, 54.9-69.4). Median OS after BM diagnosis was 9.7 months (95% CI, 5.9-13.5) in all patients (Figure 2a), and 15.6 months in the 47 patients with follow-up (95% CI, 9.8-21.3). Median OS from LMD diagnosis for the 6 patients diagnosed with LMD at the time of initial BM was 3.6 months (95% CI, 0.69-15.8). Median CNS-FFP in the 47 patients with follow-up was 18.5 months (95% CI, 9.3-27.8).

In the 28 patients with a single BM, median OS after BM was 16.1 months (95% CI, 3.8-28.3). Survival was no different in patients who received surgical resection for a single BM (log-rank, *p* = 0.32). Seven patients treated with SRS alone had median OS of 60.2 months (95% CI, 9.7-not reached).

Univariate analysis of OS included the following potential factors, including those validated in the Recursive Partitioning Analysis for brain metastases [15]: age at BM diagnosis (<65 or ≥65), primary tumor pathologic stage and grade, histology (papillary serous, endometrioid, or all others), interval from EOC diagnosis to BM, KPS at BM diagnosis (<70 or ≥70), primary tumor control status, extent of extracranial disease (limited or extensive), location of BM, number of BM, presence of LMD, surgery as part of treatment, and type of RT received (SRS, WBRT, PBRT). The following factors were significantly associated with OS on UVA: KPS less than 70 (vs ≥70, hazard ratio [HR] 2.78, *p* = 0.008), four or more BM (vs. 1–3 BM; HR, 3.75; *p* < 0.001), LMD (vs. none; HR, 5.32; *p* = 0.001), longer duration between EOC diagnosis and BM diagnosis (HR, 1.01; *p* = 0.006), uncontrolled primary tumor (HR, 2.87; *p* = 0.001), extensive extracranial metastases (vs. limited extracranial metastases; HR, 1.98; *p* = 0.036), BM in both the cerebrum and cerebellum (vs either alone; HR, 2.49; *p* = 0.003), and use of SRS (vs. WBRT or PBRT; HR, 0.46; *p* = 0.03). The use of craniotomy had no effect on OS (*p* = 0.31).

MVA (Table 2) identified KPS less than 70 (HR, 2.86; *p* = 0.018), four or more BM (vs. 1 or 2–3 BM; HR, 3.18; *p* = 0.053), LMD (vs. 1–4 BM; HR, 8.22; *p* = 0.013), and uncontrolled primary tumor (HR, 2.84; *p* = 0.008) as risk factors significantly associated with inferior OS after RT for BM. Kaplan-Meier survival curves stratified by the factors significant on MVA are shown in Figure 2b-d.

**Table 2:** Multivariate analysis of overall survival using Cox proportional hazard model

**Variable**	**Event(n)/**	**Hazard ratio**	***p *Value**
	**Total (n)**		
KPS at BM diagnosis			
≥70 (reference)	37/42	1.00	
<70	9/9	2.86	0.018
Primary tumor controlled in abdomen			
Yes (reference)	31/36	1.00	
No	23/23	2.84	0.008
Number of BM or LMD			
1 (reference)	24/28	1.00	
2-3	8/10	1.51	0.490
4+	16/16	3.18	0.053
LMD	6/6	8.22	0.013

Event = death. BM = brain metastasis; KPS = Karnofsky Performance Score; LMD = leptomeningeal disease.

**Figure 3:**
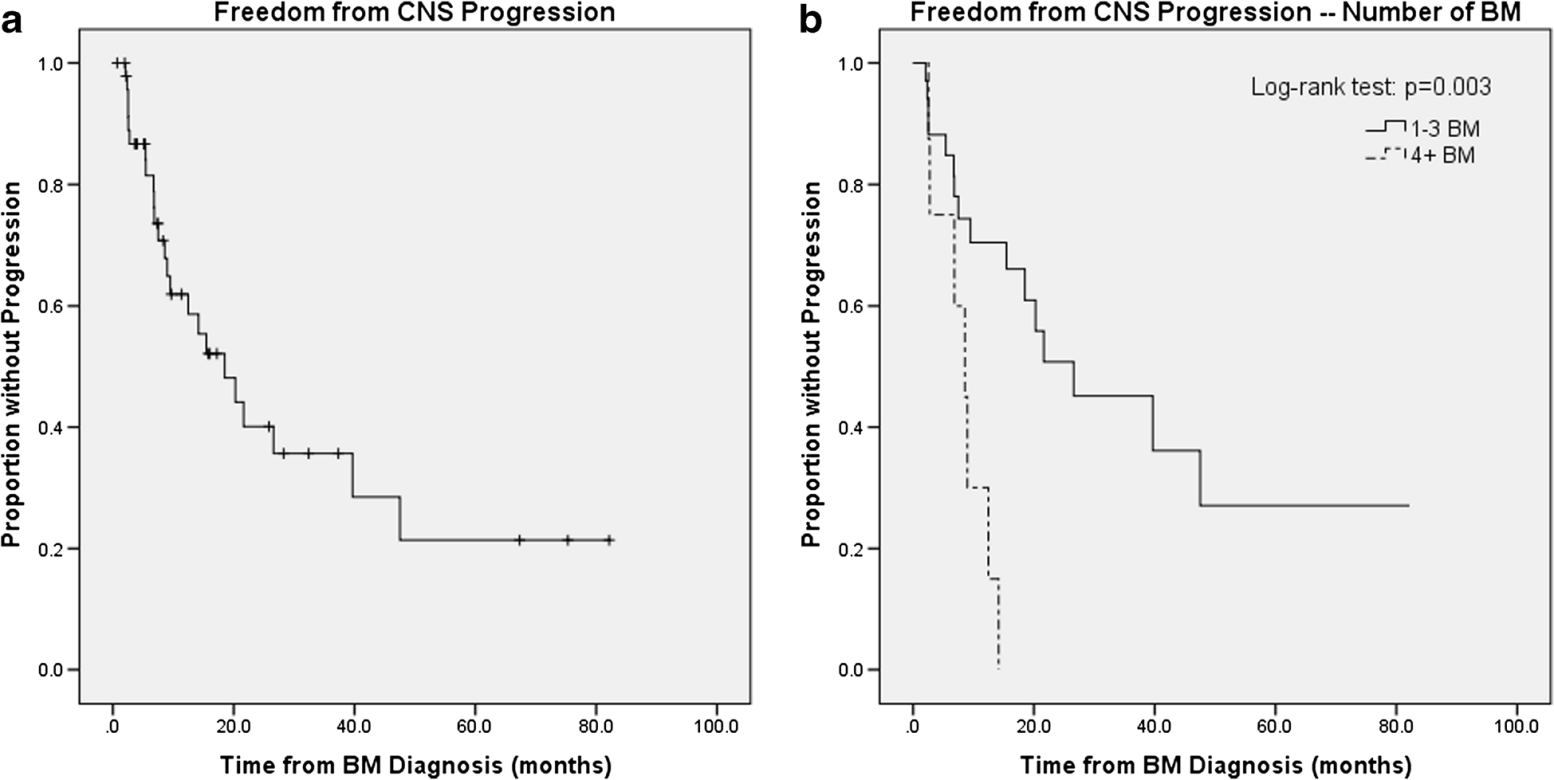
**a**. **Central nervous system (CNS) freedom from progression using Kaplan-Meier method. b**. CNS freedom from progression stratified by number of brain metastases.

On UVA for CNS-FFP, the presence of four or more BM was significant, with a negative association (HR, 4.09; *p* = 0.006), when compared with 1–3 BM (Figure 3b). In this analysis, 1 and 2–3 BM were combined since there was no significant difference in CNS-FFP between the two groups. Extensive extracranial disease was also significant (vs. limited; HR, 2.95; *p* = 0.048). On MVA for CNS-FFP, only four or more BM remained significant (HR, 2.56; *p* = 0.04),

## Discussion

Our series represents the largest in the published literature of RT for treatment of BM in EOC, with a total of 60 patients analyzed. We show that these patients can be effectively treated with RT, with or without resection of tumor, and that survival in these patients depends on KPS, number of BM, presence of LMD, and presence of uncontrolled primary tumor. We found no statistically significant effects of age, tumor histology, grade, initial disease stage, RT type, or use of surgical resection of metastases on OS after diagnosis of BM.

While CNS metastases are rare in EOC, they appear to be increasing in incidence as extracranial disease is better controlled with modern chemotherapy. In our series, patients with more advanced stage and grade comprise the majority of patients who develop BM. The most common presentation was a single BM in 47% of patients. Patients with single BM may be treated with surgery, SRS, PBRT, and/or WBRT. In our series, seven patients treated with SRS alone had a median OS of 60.2 months. While this patient subset is small, the long survival is noteworthy in a metastatic population, and likely indicates that these patients were highly selected with good control of extracranial disease and high KPS. Another smaller series has found longer survival in well-selected patients treated with SRS versus WBRT [21]. Patients treated with only WBRT tend to have shorter median survival times [22], and this may be reflective of overall poorer KPS, stage, and number of BM.

The use of surgery and RT has been associated with longer survival after BM in multiple smaller studies [3-5,8,13,23-25]; however, our study found no significant benefit to the use of surgery in 22 patients who received postoperative RT. Pothuri et al. [13] published a series from our institution of 14 patients treated with craniotomy and postoperative RT, and found median survival of 18 months and high rates of local control. Based on our current study and those preceding, we recommend that patients with a single BM, good KPS and limited extracranial disease be considered for SRS or both craniotomy and RT as clinically indicated. Ideally, single-modality SRS will be tested in more patients with EOC and single BM to come to a better understanding of the adequacy of SRS alone. In patients with multiple BM, some data suggest that resection of multiple metastases may improve outcomes [26,27]; we could not verify this finding in our population, as only two patients fell into this category.

Our results add to the findings of the Radiation Therapy Oncology Group (RTOG) Recursive Partitioning Analysis (RPA) system [15] for BM in a variety of cancers, which identified KPS ≥70, age <65 years, controlled primary carcinoma, and no extracranial systemic metastases as being predictive of the longest survival after BM. We suspect that age was not significant in our patients because the majority of our patients fell under the 65-year-old cutoff used by the RPA.

A large but older series of EOC BM includes 72 patients from MD Anderson Cancer Center [4] treated heterogeneously since 1985, and none with upfront SRS or PBRT. In that series, 8 (11%) patients received steroids alone, 35 (51%) WBRT alone, 8 (11%) surgery alone, and only 12 (17%) had both surgery and WBRT. Twenty-five patients had a single BM, and 47 had multiple. The study does not provide information on the use of salvage therapies for CNS recurrence. Their patients had inferior outcomes to those in our study; median OS was just 6.9 months in patients with a single BM. On their univariate analysis of potential prognostic factors, they did not include KPS or primary tumor control, both of which are vitally important risk factors for outcomes after BM, and are important for determining treatment approach. Although comparing outcomes between retrospective studies is challenging, several possibilities may explain why our results are superior to this series. First, almost half of our population had a single BM, while two-thirds of the patients in the MD Anderson series had multiple metastases. Second, none of our patients were treated with steroids alone, a group that had a significantly worse hazard ratio of death and accounts for 11% of their population. Third, 37% of our patients received multimodality therapy that included surgery and RT, while only 17% of patients in their study were treated in a similar fashion and represented the group with the longest median survival. Surgical resection of a single BM has been shown to improve survival in a randomized clinical trial [28]; in this MD Anderson series, it does not appear that all patients with single BM received surgery, and none received SRS. Fourth, almost two-thirds of our patients were treated with chemotherapy following treatment of their BM, which may contribute to better outcomes (although we were unable to include it in our Cox regression analysis due to its vast heterogeneity). Fifth, it is not clear that patients with relapse received salvage therapy, while most of our patients received RT or chemotherapy at CNS relapse. Lastly, patients treated at our institution are followed closely and systematically for recurrence, and are treated with early salvage therapy at time of relapse; it is unclear what the standard follow-up entailed in other studies.

Chen et al. [14] used the RTOG RPA classification system in a population of 19 patients with EOC BM treated with various approaches, and found that surgery was associated with longer OS (33.7 months vs 7.4 months, *p* = 0.006). However, only 9 patients underwent surgery, and 8 of these also received adjuvant radiotherapy to the brain, making the patient population too small to draw definitive conclusions regarding the adequacy of surgery alone for brain metastases in this population. Their UVA found primary tumor control (*p* = 0.006) and number of BM (*p* = 0.005) to be associated with OS, but the number of events was too small to perform a true multivariate analysis. In our study, we also find that primary tumor control and number of BM are important. Our patient population differs in that we have three times as many patients, and all are treated with RT. In addition, our larger patient population likely includes patients that are less highly selected than in the Chen study. In fact, Chen et al. concede in their discussion section that the relatively long overall survival of their study (median, 16.3 months) may be attributed to patient factors that are pertinent to outcome but not accounted for in the study, such as systemic therapy and institutional preference for aggressive, multimodality therapy.

A recent multi-institutional retrospective study of 139 patients with brain metastases from various gynecologic malignancies identified a group of 56 patients with ovarian, fallopian tube, or peritoneal histology [11] (it is unclear how many of these patients had a true epithelial ovarian carcinoma). While comparing this “ovarian” subgroup to our patients is not entirely possible, given the heterogeneity of their subgroup, the study has several noteworthy findings. First, in the “ovarian” group, 80% received RT, about half of which also received surgery and/or chemotherapy for the BM. Second, median survival for the 56 patients was 12.5 months. Third, on a multivariate analysis across all gynecologic types, they found ovarian/tubal/peritoneal disease origin was associated with improved survival and recommended these patients be treated more aggressively when a BM is diagnosed.

In our series, 10 (20%) patients with BM also had LMD, and 9/10 (90%) had a synchronous or prior BM. LMD is an uncommon occurrence in advanced malignancy, estimated to occur in 4-15% of solid tumors [19]. Previous series have reported an incidence of synchronous or preexisting CNS metastases in 28-75% of patients with LMD [17]. Clarke et al. [18] reviewed a large series of LMD from our institution and reported that 70% of patients with LMD from solid tumors had previous or current brain disease. In that study, 59% of patients were treated with RT, 15% received supportive therapy only, and the remainder had some form of chemotherapy. Median OS after LMD was 2.3 months for patients with solid tumors; there were 2 cases of ovarian carcinoma. Another large series of 155 patients with LMD treated from 1980 to 2002 found a median OS in solid tumors of 2.8 months in non-breast solid tumors [29]. Only one patient had ovarian cancer.

We found that median OS after LMD when present at initial BM diagnosis (n = 6) was 3.55 months (95% CI, 0.69-15.8). The patients in our series who developed LMD after a preceding BM diagnosis had no higher representation of one primary treatment modality, either surgery or RT, prior to developing LMD. Our patient population is different from these other large studies for several reasons. First, LMD was primarily treated with RT in our study, in contrast to other reports that frequently use intrathecal or systemic chemotherapy as the principal treatment for LMD [30,31]. In one series of 31 patients treated with intrathecal chemotherapy, the response rate was 52% [32]. In contrast, our LMD patients treated with RT who had follow-up imaging (n = 6) demonstrated 100% overall response rate (both complete and partial response) as seen on their first follow-up MRI. Second, all patients had LMD diagnosed by MRI, with or without CSF analysis. Lastly, our study represents a homogenous population of one disease, as opposed to the existing series that include one or two ovarian cancers mixed in with various other solid tumor types. Based on the favorable responses to RT and slightly longer survival in our series, we support administering RT for EOC LMD. EOC is known to be a biologically radiosensitive disease, and our results in the setting of LMD appear to confirm this fact.

There are some limitations of our study. First, we performed a retrospective analysis, which has its own well-known inherent drawbacks and biases, many of which have been outlined above. Second, not all patients in our study had pathologic confirmation of BM from EOC, which could theoretically have led us to include patients with primary CNS tumors or benign disease in this cohort. Third, because it was difficult to determine cause of death for many patients in this retrospective analysis, we could not determine BM-specific survival, which would be a relevant measure of outcome in this population. Lastly, while we were able to determine which patients received any systemic chemotherapy before and after RT for BM, we were unable to routinely quantify the number of cycles of chemotherapy they received and the specific agents with which they were treated. Prospectively collected data on chemotherapy is required to draw strong conclusions about the efficacy of systemic therapy in this patient population.

## Conclusions

This study is the largest reported series of patients with EOC BM treated with modern radiation therapy. We show that KPS less than 70, four or more BM, leptomeningeal disease, and uncontrolled primary tumor predict for inferior survival after radiation therapy for EOC BM. A single BM is the most common presentation, occurring in 47% of patients, and is associated with longer survival when treated with SRS or surgery + RT. Advanced-stage EOC is associated with shorter median interval to BM. Over half of patients with long-term follow-up will recur in the CNS, but can be salvaged effectively with RT or surgery. LMD is associated with poor survival and occurs in 20% of our patients. RT can result in partial or complete response of LMD, although patients will likely recur or progress.

## Abbreviations

BM: Brain metastases; LMD: Leptomeningeal disease; EOC: Epithelial ovarian cancer; RT: Radiation therapy; KPS: Karnofsky performance status; CNS: Central nervous system; WBRT: Whole brain radiation therapy; PBRT: Partial brain radiation therapy; SRS: Stereotactic radiosurgery; OS: Overall survival; PFS: Progression free survival; FFP: Freedom from progression; MVA: Multivariate analysis; UVA: Univariate analysis; HR: Hazard ratio; RPA: Recursive partitioning analysis

## Competing interests

The authors have no financial competing interests and no non-financial competing interests.

## Authors’ contributions

ST, KA, KB made substantial contributions to conception and design and analysis and interpretation of data. ST and VM performed all acquisition of data. All authors (ST, VM, KA, KB, MH, VT, and CA) were involved in drafting the manuscript and revising it critically for important intellectual content. All authors read and approved the final manuscript.

## Authors’ information

CA is Chief of the Gynecologic Medical Oncology Service at MSKCC. KA is Member, Memorial Sloan-Kettering Cancer Center (MSKCC) and Professor of Radiation Oncology. VT is Associate Professor of Neurosurgery at MSKCC. MH is Associate Professor of Medicine and part of the Gynecologic Medical Oncology Service. VM is Assistant Professor of Medicine and part of the Gynecologic Medical Oncology Service. KB is Assistant Professor of Radiation Oncology and the principal CNS Radiation Oncologist at MSKCC. ST is chief resident in the Department of Radiation Oncology.

## 

Data presented in poster form at the American Society for Therapeutic Radiology and Oncology Annual Meeting, October 2–4, 2011, Miami, FL (abstract #2568).
